# Has the increased participation in the national campaign ‘Dry January’ been associated with cutting down alcohol consumption in England?

**DOI:** 10.1016/j.drugalcdep.2021.108938

**Published:** 2021-10-01

**Authors:** Philippa Case, Colin Angus, Frank De Vocht, John Holmes, Susan Michie, Jamie Brown

**Affiliations:** a Department of Epidemiology & Public Health, University College London, WC1E 7HB, UK; b Sheffield Alcohol Research Group, School of Health and Related Research, University of Sheffield, UK; c Population Health Sciences, University of Bristol, UK; d NIHR Applied Research Collaboration West (ARC West), UK; e Department of Clinical, Educational and Health Psychology, University College London, UK; f Department of Behavioural Science and Health, University College London, UK

**Keywords:** Dry January, temporary abstinence, alcohol, media campaigns, harm reduction

## Abstract

**Aims:**

Dry January is a national multimedia campaign in the UK that encourages people to abstain from drinking alcohol during the month of January. The population-level campaign makes extensive use of email and social media to support participants and has reported a substantial increase in participation since 2015. This study aimed to assess whether the increase in participation in Dry January between 2015 and 2018 was associated with reduced alcohol consumption in England.

**Design:**

Repeat cross-sectional design.

**Setting:**

England, March 2014 to January 2018.

**Participants:**

A total of 37,142 respondents to the Alcohol Toolkit Study, a monthly in-home survey of alcohol consumption among representative cross-sectional samples of people aged 16+ years in England.

**Measures:**

Outcomes included i) percentage of adults reporting drinking monthly or less frequently in the last 6 months and ii) mean weekly alcohol consumption among drinkers derived from the Alcohol Use Disorders Identification Test questions on typical frequency and quantity in the last 6 months.

**Analyses:**

For each outcome, regression models were fitted for month: January (2015 and 2018) vs March-December (2014 and 2017) and for year: 2014/15 vs 2017/18. Interaction terms were included in the models to examine whether the difference between January and the preceding months on each outcome measure depended upon the year (2014/15 vs 2017/18). For non-significant interactions, Bayes factors were calculated to assess the relative strength of evidence for large effects (OR = 1.80 on monthly drinking and β=-1.0 on mean consumption) compared with the null.

**Results:**

Differences between January and other months were similar in 2014/15 and 2017/18 for adults reporting drinking monthly or less frequently and the mean consumption among drinkers (OR = 0.91, 95 %CI 0.79–1.05, BF = 0.05; β = 0.55, 95 %CI=-0.14 to 1.25, BF = 0.13 respectively).

**Conclusions:**

The increase in participation in Dry January between 2015 and 2018 was not associated with large corresponding changes in people drinking monthly or less frequently over the last 6 months, or in mean weekly consumption among drinkers.

## Highlights

• Dry January was not associated with large population-level declines in alcohol consumption.
• People were more likely to cite Detox (e.g., Dry January) as a motive in a quit attempt in January.
• The proportion of people citing Detox (e.g., Dry January) throughout the year as a reason for a quit attempt increased between 2014 and 2018.

## 1 Introduction

Dry January is an annual alcohol abstinence initiative run by Alcohol Change UK (formerly Alcohol Concern), which encourages people to abstain from drinking alcohol for the month of January. It aims to reduce alcohol consumption by enabling people to ‘take control’ of their drinking, which includes reducing the quantity and frequency of drinking, and by encouraging conversations by the public about reducing alcohol consumption (Alcohol Concern, 2017a).

Dry January launched in 2013 in the UK and has gained popularity (de Visser et al., 2017). Alcohol Change UK report that 4000 took part in the first campaign while in 2018 an estimated 4 million participated with 40,000 people signing up to participate via the Dry January app or via email to receive daily email updates and support (Alcohol Change UK, 2019). People who sign up to participate in Dry January can use the app to track units, calories and money saved and/or can receive daily emails with supportive stories and tips. There is also support available via the campaign website and social media platforms. Whilst those who sign up officially are more likely to remain abstinent for the entire month (de Visser et al., 2017; Alcohol Change UK, 2021), official participation only accounts for 1% of participation in Dry January (Alcohol Change UK, 2019) and it is therefore unofficial participation that is of interest at a population level.

Increased participation has been accompanied by an increase in media coverage and social media engagement, which has been attributed to Public Health England’s collaboration with Alcohol Concern in 2015 to enhance diffusion of the campaign (de Visser et al., 2017; Alcohol Concern, 2017b). This upward trend has been reflected in Google searches for Dry January, which were 4.5 times higher in January 2018 compared with January 2013 (Google, 2021). To encourage people to participate, the Dry January campaign promotes the short-term benefits to physical and mental health, as well as long-term reductions in alcohol consumption for Dry January participants (Alcohol Change UK, 2021). Although Dry January does not set explicit goals for behaviour change against which the campaign can be evaluated, in view of its popularity and to support decisions on whether to invest public funds into the campaign, there is a need to establish whether the growth of Dry January has translated into reduced alcohol consumption that is detectable at the population-level.

There are several initiatives involving short-term alcohol abstinence (Febfast, 2021; Dry July Foundation, 2019; Cancer Research UK, 2021; Macmillan Cancer Support, 2021; Bartram et al., 2018), however, few quantitative studies of their effectiveness have been conducted. In the UK, a prospective longitudinal study of Dry January participants published in 2016 (De Visser et al., 2016) found that people who successfully completed Dry January reported significant reductions in drinking days per week (*Cohen’s d* = .53), drinks per typical drinking day (*d* = .25) and frequency of drunkenness (*d* = .40) 6-months after the end of Dry January. However, the authors found similar reductions for participants who did not complete Dry January. An evaluation of Febfast 2011 in Australia suggested participants reduced their alcohol consumption compared with the general population in the short-term (Hillgrove and Thomson, 2012).

A qualitative study exploring the appeal of Dry January and possible mechanisms of action, found that participants reported benefits to physical and mental health and well-being (Yeomans, 2018). By focusing on the immediate benefits of abstaining from alcohol, participants are not required to prioritise future health over present enjoyment, making Dry January stand out from traditional public health campaigns. Dry January may have also helped to shift cultural and social norms around drinking (Yeomans, 2018), leading to improved engagement in non-alcohol focused leisure activities such as sport (Bartram et al., 2017; Fry, 2011), and empowerment to participate in social situations without drinking alcohol (De Visser et al., 2016).

January was chosen by Alcohol Change UK due to the benefits of having a break from alcohol following Christmas, a traditionally heavy drinking period (Alcohol Change UK, 2019). There is a longstanding trend of reduced alcohol consumption in January evidenced by an annual pattern of reduced alcohol duty income (HM Revenues and Customs, 2019) and reduced alcohol sales (Knudsen and Skogen, 2015). This is supported by evidence from Europe that consumption varies by season, typically peaking in summer and slumping during winter (except the Christmas period) with January being a particularly cold month in the UK (Knudsen and Skogen, 2015; Uitenbroek, 1996; Lemmens and Knibbe, 1993). A recent study found a moderate increase in attempts to reduce alcohol consumption in January compared with other months in England, although this was not accompanied by a decrease in reported alcohol consumed (De Vocht et al., 2016). Further work is required to establish whether the apparent increased participation in Dry January in recent years has affected January alcohol consumption and attempts to reduce consumption at a population-level, or whether participation in Dry January reflects a re-organisation of the long-standing pattern of reduced drinking in winter months, particularly in January in the UK. This study addressed the following research question:

Has the reported increased participation in Dry January between 2015 and 2018 been associated with alcohol reduction activities independently of pre-existing seasonal variation?

## 2 Methods

### 2.1 Study design

The Alcohol Toolkit Study (ATS) is a monthly, cross-sectional, nationally representative in-home survey of adults aged 16+ in England living in private households. Data on the prevalence of at-risk drinking, at-risk drinker attempts to reduce consumption, methods used to reduce consumption and the receipt of advice from healthcare professionals are collected alongside sociodemographic and smoking information (Beard et al., 2015). This study examined whether the difference between January and the preceding months on alcohol reduction outcomes in the English adult population depended upon the year (2015 vs 2018).

### 2.2 Participants

Each month, approximately 1700 adults aged 16+ in England are interviewed. Interviews are conducted mid-month (weeks two or three). Sampling is a hybrid between random location and quota sampling, details of which can be found in the ATS protocol (Beard et al., 2015). This study used data from March 2014 to January 2015 (the first January of data collection: N = 18,543 unweighted) and March 2017 to January 2018 (the most recent year of data at the time of analysis: N = 18,752 unweighted).

### 2.3 Measures

#### 2.3.1 Explanatory

(i) Month: Data collection months were dichotomised into January vs. non-January (March-December of the preceding year). February was excluded to mitigate the effect of participants reporting January activity in February when such activities may still be salient. Whilst this may result in December’s activity being reported in January, using January data was still considered the best way of assessing January activity.
(ii) Year: Data collection year was dichotomised into 2014/15 (Dry January 2015) and 2017/18 (Dry January 2018).

#### 2.3.2 Outcomes related to alcohol consumption

The ATS includes the 3-item consumption questions from the Alcohol Use Disorders Identification Test (AUDIT-C) (Babor et al., 2001; Conigrave et al., 1995; Bohn et al., 1995). The questions are framed in terms of ‘*alcohol you have drunk in the last 6 months*’ but have demonstrated responsiveness to short-term changes (Bush et al., 1998; Bradley et al., 1998). This assessment of the association between increased participation in Dry January and consumption outcomes relies on this responsiveness.

(i) *Percentage of all adults reporting drinking monthly or less frequently*.

This was assessed using AUDIT-C question 1: *‘How often do you have a drink containing alcohol?*: 1 *(Never)*, 2 *(Monthly or less)*, to 6 *(6 or more times per week)’.* Scores of 1 and 2 were recoded as ‘monthly or less frequently’.

(ii) *Mean weekly alcohol consumption among adults who report drinking*.

This outcome was derived from AUDIT-C questions 1: *‘How often do you have a drink containing alcohol?*’ and 2: *‘How many standard drinks containing alcohol do you have on a typical day when you are drinking?*: 1 *(1-2)* to 7 *(16+)’*. One standard drink was equivalent to one UK alcohol unit and the number of units contained in a range of drinks was included to help participants calculate their consumption. In line with previous research (Beard et al., 2019), mid-range scores from items 1 and 2 were multiplied to calculate average weekly consumption. For example, if a respondent consumed alcohol 4-5 times/week (AUDIT-C 1) and consumed 7-9 standard drinks/typical drinking day (AUDIT-C 2), mean weekly consumption was calculated as 4.5 × 8 = 36. Where participants reported drinking 16+ units on a typical drinking day, 16 was used due to the absence of a mid-point.

#### 2.3.3 Outcomes related to attempts to restrict alcohol consumption

We planned to report outcomes related to attempts to cut down, which are assessed only among at-risk drinkers (classified as scoring ≥5 on the AUDIT-C). These outcomes could only reflect increased participation in Dry January if either i) Dry January affected attempts but did not translate to changes in consumption or ii) the consumption questions were not sufficiently responsive to short-term change. Otherwise, recent consumption changes would lead to Dry January participants not being classified as at-risk drinkers. Therefore, we included the following additional measures to assess the effects of Dry January under the alternative assumption that consumption did not change or that consumption changes could not be detected using AUDIT-C.

(i) *Percentage of at-risk drinkers reporting a current attempt to restrict alcohol consumption.*

This outcome was assessed by the question ‘*Are you currently trying to restrict your alcohol consumption? (Yes/no)’*.

(ii) *Percentage of at-risk drinkers citing Detox (e.g., Dry January) as a motive in their most recent attempt to restrict alcohol consumption.*

Those reporting an attempt to restrict alcohol consumption in the past 12 months (including current attempts) were asked: ‘*Which of the following, if any, do you think contributed to you making the most recent attempt to restrict your alcohol consumption?’*

(iii) *Percentage of at-risk drinkers reporting use of a website or app to help to restrict alcohol consumption in their most recent attempt.*

Those reporting an attempt to restrict alcohol consumption in the past 12 months (including current attempts) were asked *‘Which, if any, of the following did you use to try to help restrict your alcohol consumption during the most recent attempt?’* With the option: *‘Visited a website for help with drinking/used an alcohol application (‘app’) on a handheld computer…)’*

### 2.4 Analysis

Logistic regression analyses with interaction terms were conducted with the dichotomous outcomes (percentage of all adults reporting drinking monthly or less frequently, percentage of at-risk drinkers reporting a current attempt to restrict alcohol, percentage of at-risk drinkers citing Detox (e.g. Dry January) as a motive in their most recent attempt to restrict alcohol consumption, percentage of at-risk drinkers reporting us of a website or app to help restrict alcohol consumption in their most recent attempt) as the dependent variables, and a linear regression with the continuous consumption outcome (mean weekly alcohol consumption) as the dependent variable. Month (Jan vs Mar-Dec) and year (2017/18 vs 2014/15) were the independent variables. Where interaction effects were identified, the analysis was stratified by year to estimate the simple effect. Bayes factors were calculated to explore whether non-statistically significant findings could be better explained by data that were insensitive (i.e. unable to differentiate between the hypothesis and the null hypothesis) or whether the data supported the null hypothesis of no interaction between year and month.

Data were weighted using a rim (marginal) weighting technique to match the English population. The validity of the weighting was explored in a sensitivity analysis that repeated the analysis on unweighted data adjusted for socio-demographic characteristics (sex, age, and region). The pattern and direction of results were largely similar for analyses of weighted and unweighted data.

All analyses excluding the calculation of Bayes factors were conducted in STATA 15.0. Bayes factors were produced using an online calculator (http://www.lifesci.sussex.ac.uk/home/Zoltan_Dienes/inference/Bayes.htm).

All analyses were preregistered on OpenScienceFramework: osf.io/35h4e. In our pre-registered plan, we listed the outcomes in a different order, including two primary and three secondary outcomes. In writing the discussion, it became apparent that different outcomes made different assumptions about the responsiveness of the AUDIT-C, as described above.

## 3 Results

### 3.1 Percentage of adults reporting drinking monthly or less frequently

**Fig. 1:**
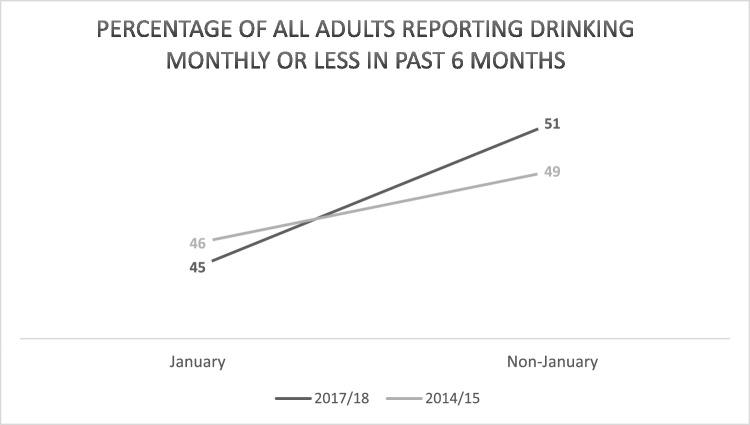
Percentage of all adults reporting drinking monthly or less frequently in past 6 months (weighted).

The percentage of adults drinking monthly or less frequently was lower in January than the preceding March-December in both 2014/15 (46 % vs 49 %) and 2017/18 (45 % vs 51 %; Table 1; Fig. 1; the monthly time-series is presented in Figure S1 of the supplementary data files). The odds of reporting drinking monthly or less frequently were significantly lower in January vs non-January (OR = 0.84, 95 %CI 0.79−0.91) and significantly higher in 2017/18 vs 2014/15 (OR = 1.06, 95 %CI 1.02–1.10). The interaction between month and year was non-significant (OR = 0.91, 95 %CI 0.79–1.05) with a Bayes factor of 0.05 indicating support for the null hypothesis of no interaction. This implies that the odds of reporting drinking monthly or less frequently in January vs non-January months did not differ significantly between 2017/18 and 2014/15.

**Table 1:** Percentage of all adults reporting drinking monthly or less frequently in the last 6 months.

	All drinkers	
	Drinking monthly or less frequently	Drinking more than monthly
January 2015		
Unweighted N (%)	804 (48)	861 (52)
Weighted N (%)	766 (46)	901 (54)

March-December 2014		
Unweighted N (%)	8706 (52)	8077 (48)
Weighted N (%)	8227 (49)	8562 (51)

January 2018		
Unweighted N (%)	739 (43)	967 (57)
Weighted N (%)	772 (45)	967 (55)

March-December 2017		
Unweighted N (%)	8549 (50)	8439 (50)
Weighted N (%)	8599 (51)	8391 (49)

Total		
Unweighted	18,798	18,334

Considering the choice to use ‘monthly or less frequently’ rather than ‘never’, an unplanned sensitivity analysis was conducted using ‘never’ as the outcome and this did not alter the pattern of results. In case reporting in January was not responsive to changes in drinking started in January, this analysis was repeated using February vs preceding months, which showed the same pattern of results. All sensitivity analyses are reported under supplementary data.

### 3.2 Mean weekly alcohol consumption among adults who report drinking

Mean weekly consumption ranged from 5.2 units (SD 8.8) (January 2015) to 5.7 units (SD 10.3) (January 2018; Table 2; the monthly time-series is presented in Figure S2 of the supplementary data files). Mean consumption did not differ significantly between January and non-January months (β = 0.23, 95 %CI=-0.11 to 0.58) or between 2017/18 and 2014/15 (β=-0.003, 95 %CI=-0.20 to 0.20). The interaction between month and year was non-significant (β = 0.55, 95 %CI=-0.14 to 1.25). The Bayes factor was 0.13, indicating support for the null hypothesis of no interaction between month and year.

**Table 2:** Mean weekly alcohol consumption among adults who report drinking.

	All drinkers	
	Mean weekly consumption in units	No. observations
January 2015		
Unweighted (SD)	5.3 (9.4)	1661
Weighted (SD)	5.2 (8.8)	1661

March-December 2014		
Unweighted (SD)	5.1 (10.0)	16,732
Weighted (SD)	5.3 (9.8)	16,732

January 2018		
Unweighted (SD)	6.2 (11.1)	1706
Weighted (SD)	5.7 (10.3)	1706

March-December 2017		
Unweighted (SD)	5.3 (10.0)	16,960
Weighted (SD)	5.2 (9.9)	16,960

Total		
Unweighted		37,059

### 3.3 Percentage of at-risk drinkers reporting a current attempt to restrict alcohol consumption

Having established no large association (where a large effect was considered to be OR = 1.80 on monthly drinking and β=-1.0 on mean consumption) with the outcomes related to alcohol consumption, it is useful to assess the extent to which there was an association between activities among at-risk drinkers, which may not have translated to changes in consumption (De Vocht et al., 2016).

**Fig. 2:**
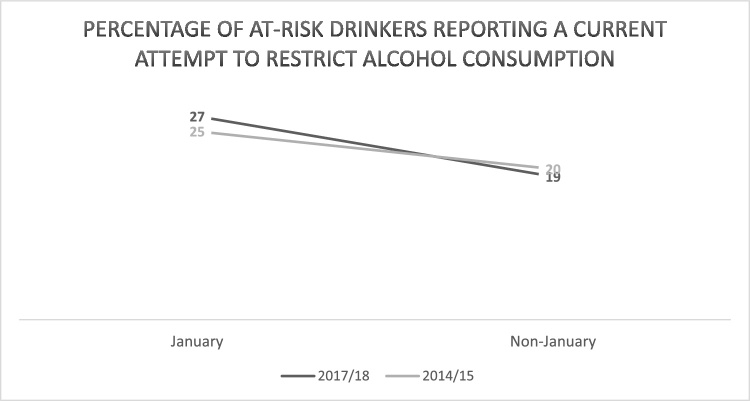
Percentage of at-risk drinkers reporting a current attempt to restrict alcohol consumption (weighted).

Of the total sample, 26 % were at-risk drinkers (scoring ≥5 on AUDIT-C). The percentage of at-risk drinkers reporting a current attempt to restrict consumption was higher in January than non-January months in both 2014/15 (25 % vs 20 %) and 2017/18 (27 % vs 19 %; Table 3; Fig. 2). The odds of at-risk drinkers reporting a current attempt to restrict consumption were significantly higher in January vs non-January (OR = 1.46, 95 %CI 1.25–1.70). There was no significant difference by year (2017/18 vs 2014/15) (OR = 0.92, 95 %CI 0.84–1.02). The interaction between month and year was non-significant (OR = 1.21, 95 %CI 0.89–1.65). The Bayes factor was 0.88, indicating that the data were insensitive and could not distinguish between no effect and an interaction between month and year.

**Table 3:** Number of at-risk drinkers reporting a current attempt to restrict alcohol consumption; number citing detox (e.g., Dry January) and number using a website/app in most recent attempt to restrict consumption.

	At-risk drinkers (participants scoring ≥5 on AUDIT-C)					
	Current attempt to restrict alcohol consumption		Citing *Detox (e.g., Dry January)* as motive in most recent attempt to restrict consumption		Use of website or app to help restrict alcohol consumption in most recent attempt	
	Yes	No	Yes	No	Yes	No
January 2015						
Unweighted N (%)	88 (20)	342 (80)	11 (9)	106 (91)	1 (1)	116 (99)
Weighted N (%)	112 (25)	330 (75)	18 (13)	120 (87)	2 (2)	136 (98)

March-December 2014						
Unweighted N (%)	845 (20)	3366 (80)	36 (4)	952 (96)	20 (2)	969 (98)
Weighted N (%)	915 (20)	3553 (80)	40 (4)	1024 (96)	21 (2)	1045 (98)

January 2018						
Unweighted N (%)	130 (26)	369 (74)	28 (17)	139 (83)	4 (2)	163 (98)
Weighted N (%)	135 (27)	363 (73)	30 (18)	137 (82)	4 (2)	164 (98)

March-December 2017						
Unweighted N (%)	853 (19)	3623 (81)	128 (10)	1112 (90)	37 (3)	1203 (97)
Weighted N (%)	869 (19)	3744 (81)	137 (11)	1126 (89)	39 (3)	1225 (97)

Total						
Unweighted	1924	7719	203	2319	62	2461
Total no. respondents per outcome	9643		2522		2523

### 3.4 Percentage of at-risk drinkers citing Detox (e.g., Dry January) as a motive in their most recent attempt to restrict alcohol consumption

**Fig. 3:**
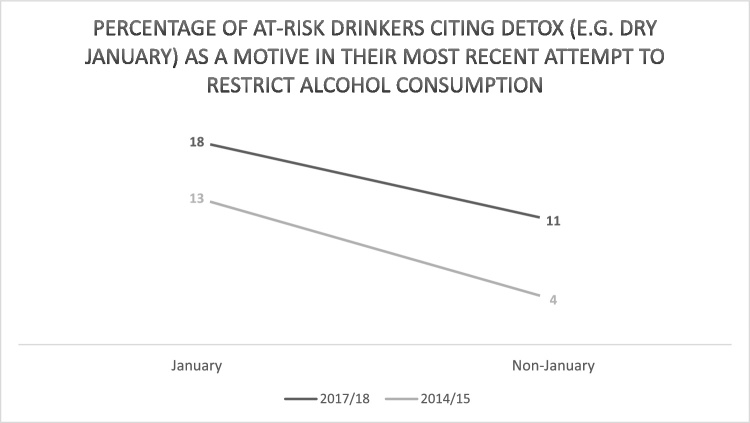
Percentage of at-risk drinkers citing *Detox (e.g., Dry January)* as a motive in their most recent attempt to restrict alcohol consumption (weighted).

The percentage of at-risk drinkers citing *Detox (e.g., Dry January)* as a motive in their most recent attempt to restrict consumption was higher in January than non-January months in both 2014/15 (13 % vs 4%) and 2017/18 (18 % vs 11 %; Table 3; Fig. 3). The odds of at-risk drinkers citing *Detox (e.g., Dry January)* as a motive in their most recent attempt to restrict consumption were significantly higher in January vs non-January (OR = 2.29, 95 %CI 1.62–3.22) and significantly higher in 2017/18 vs 2014/15 (OR = 2.59, 95 %CI 1.90–3.53). The interaction between month and year was significant (OR = 0.47, 95 %CI 0.23−0.97), with the odds of citing *Detox (e.g., Dry January)* being significantly higher in January vs non-January in 2014/15 (OR = 3.86, 95 %CI 2.15–6.92) vs 2017/18 (OR = 1.81, 95 %CI 1.18–2.79).

### 3.5 Percentage of at-risk drinkers reporting use of a website or app to help restrict alcohol consumption in their most recent attempt

The percentage of at-risk drinkers reporting use of a website/app to help in their most recent attempt was the same in January vs non-January months in 2014/15 (2%) and similar in January vs non-January in 2017/18 (2% vs 3%: Table 3). There were no significant differences by month (January vs non-January) (OR = 0.77, 95 %CI 0.33–1.78) or year (2017/18 vs 2014/15) (OR = 1.57, 95 %CI 0.94–2.61). The interaction between month and year was non-significant (OR = 0.75, 95 %CI 0.13–4.23). The Bayes factor was 0.73, indicating an insensitive result.

In order to explore any trends, planned sensitivity analyses comparing 2015/16 and 2016/17 vs 2014/15 were conducted and are reported under supplementary data.

## 4 Discussion

The increased participation in Dry January between 2015 and 2018 was not associated with corresponding large changes in people drinking monthly or less frequently over the last 6 months, or in mean weekly consumption among drinkers (where a large effect was considered to be OR = 1.80 on monthly drinking and β=-1.0 on mean consumption). The data were insensitive on whether there were large corresponding changes in reported attempts to cut down consumption among at-risk drinkers or reported use of a website or app to support recent attempts to restrict consumption. The odds of citing Detox (e.g., Dry January) as a motive in a recent quit attempt were greater in January than other months but the difference was **smaller** in 2017/18 than in 2014/15, which is inconsistent with a large increase in participation.

Previous studies of Dry January and other alcohol abstinence initiatives have indicated participation may be associated with reduced consumption (De Visser et al., 2016; Hillgrove and Thomson, 2012). These observational studies could not establish whether people may have reduced their consumption regardless of their participation. In the current study, increased participation in Dry January was not associated with large declines in alcohol consumption detectable at the population-level. Instead, the widely reported increased participation may have resulted from a re-organisation of long-standing reduction activity in January linked to the winter months and post-Christmas period in the UK (i.e., people that participate would have attempted to cut down their drinking during January regardless of the campaign).

Similar to previous studies, attempts to reduce alcohol consumption were higher in January compared to other months (De Vocht et al., 2016). The increase in attempts to cut down in January across all years did not appear to be matched by a reduction in reported mean units consumed per week. This corresponds with findings from previous research (De Vocht et al., 2016), which may reflect motivation to cut down being insufficient to cause measurable changes in consumption. Participants were also more likely to report drinking monthly or less frequently in more recent years, reflecting the recent downward trend in consumption amongst some groups (Oldham et al., 2018).

Amongst respondents who reported an attempt to restrict alcohol consumption in the past 12 months, the odds of citing *Detox (e.g. Dry January)* as a motive was greater in January compared with other months as expected. However, the association was greater in the 2014/15 (when Dry January was said to have had lower participation) than in the most recent year when participation in Dry January was reportedly higher. This seemed to reflect the proportion of people citing *Detox* in the rest of the year growing more quickly (4 % in 2014/15 compared with 11 % in 2017/18) than it did in January (13 % vs 18 %). This may be a spill over effect from increased coverage of Dry January, Dryathlon and Go Sober for October leading people to ‘detox’ throughout the year. One possible mechanism is through people being empowered to stop drinking for periods throughout the year (in line with de Visser et al.’s findings around increased drink refusal self-efficacy (De Visser et al., 2016)). Alternatively, it may be a more general phenomenon with detox being widely promoted by public discourse around health and wellness. This paper has explored the impact of Dry January on January drinking outcomes, but it is possible that the campaign (and other similar campaigns) may have had a broader systems effect by changing culture and affecting long-term and overall consumption, which we have not been able to evaluate here. Future research might explore how these initiatives have impacted alcohol reduction activities more broadly via increased acceptability of abstinence or might take a complex systems perspective (McGill et al., 2021, 2020) to explore how the broader system in which campaigns such as Dry January take place may interact with any changes driven by such campaigns.

### 4.1 Limitations

Alcohol consumption was measured using the self-reported AUDIT-C, which asked respondents to consider their consumption in the last six months. However, the AUDIT-C is a validated tool and responsive to short-term change (Bradley et al., 1998; Greenfield and Kerr, 2008). Our finding that drinking more than monthly over the last six months was significantly greater in January suggests the responsiveness was limited. Instead, the January reporting likely reflected the increases in drinking typically found over the Christmas period (Knudsen and Skogen, 2015; Uitenbroek, 1996; Cho et al., 2001). Conversely, the sensitivity analysis indicated no difference in February compared with other months. If the measure had been reflecting drinking over the last six months, the increase from December should still have been evident in February. Taken together, this suggests the AUDIT-C is responsive to change over the last month or so, rather than reflecting immediate change. Critically, in both the main and sensitivity analyses, regardless of the absolute difference between January (or February) and other months, the increased participation should have reduced this difference between years. However, there was evidence that the expected large interaction was not detectable at the population-level.

Another limitation is that attempt-related outcomes were *only* assessed in at-risk drinkers. If Dry January caused people who would have been classified as at-risk drinkers in December to substantially reduce their consumption, then a proportion of these people would not have been classified as at-risk drinkers when surveyed in January and their attempts would not have been assessed. However, the current study found evidence of no association between increased participation and large reductions in consumption, nor did it find large reductions in consumption reported in January across all years, suggesting it is unlikely a large number of Dry January participants were missed.

A further limitation is that mean consumption was estimated among drinkers only. If a large proportion of people who drank heavily before January reported non-drinking in our January surveys, then mean consumption among drinkers – rather than all people – would underestimate the impact of Dry January. We considered adding a sensitivity analysis assessing consumption among all adults. However, having established that the proportion of non-drinkers did not increase in January – and in fact decreased – it was judged unnecessary because this decrease suggests any underestimation was unlikely.

The ATS did not collect data before the first Dry January campaign and it was therefore only possible to compare data from two time points at which Dry January was thought to have lower (2014/15) and higher (2017/18) participation. Therefore, findings do not relate to any overall impact of Dry January.

The measure relating to whether people cited ‘*Detox (e.g. Dry January)*’ included people undertaking a detox for any reason. A change in the focus of Dry January away from ‘detox’ over time would have undermined the ability to detect a true increase in Dry January being cited as a motive. However, we are not aware of such a focus shift.

The analysis did not take account of any UK policy changes within the period studied; however, we would argue that has been very little substantive change to UK alcohol policy within this period.

The measures relating to detox and use of a website or app referred to current or most recent attempt in the last 12 months. This means that the reports in January may have related to activity from previous months. Although this would have reduced the sensitivity of the measure, everything else being equal, increases in January over and above other months, should still have been detected by this measure.

Finally, whilst we consider a population-level change in alcohol a key indicator of the success of the national Dry January campaign, such changes are not necessarily the outcomes by which the campaign organisers would judge success.

### 4.2 Conclusion

The increase in participation in Dry January between 2015 and 2018 was not associated with a large corresponding reduction in alcohol consumption in England. Among at-risk drinkers, the data were insensitive on whether the reported increased participation in Dry January was associated with large increases in the percentage reporting attempts to cut down consumption in January compared with other months, or with reported use of a website or app to support their most recent attempt to cut down. There was an increase – not limited to January – in those who cited *Detox (e.g. Dry January)* as a motive between 2015 and 2018 but the increased odds of citing the motive in January compared with non-January months was greater in 2014/15 than in 2017/18, which is inconsistent with a large increase in participation. Future evaluations might take a broader systems perspective (e.g. (McGill et al., 2021, 2020) to evaluate the Dry January campaign in the context of system-level determinants of alcohol consumption.

## Role of funding source

The Alcohol Toolkit Study receives funding from the 10.13039/501100012618NIHR School for Public Health Research (SPHR1 and 2). SPHR is a partnership between universities. Data collection for the Alcohol and Smoking Toolkit Studies were supported by 10.13039/501100000289Cancer Research UK (C1417/A22962). The views expressed are those of the authors(s) and not necessarily those of the NHS, NIHR, or Department of Health. No funders had any involvement in the design of the study, the analysis or interpretation of the data, the writing of the report, or the decision to submit the paper for publication. JB is a member of the SPECTRUM consortium, supported by the UK Prevention Research Partnership (MR/S037519/1). PC is funded by the Medical Research Council’s Doctoral Training Programme.

## Contributors

PC led on creating the protocol, completing the analysis and writing the first draft on the manuscript with support from JB. All named authors were involved in the development of the research protocol and in reviewing and amending the manuscript.

## Declaration of Competing Interest

The authors report no declarations of interest.

## Footnotes

Appendix A Supplementary material related to this article can be found, in the online version, at doi:https://doi.org/10.1016/j.drugalcdep.2021.108938.
