# Supplementary material for: Has the increased participation in the national campaign ‘Dry January’ been associated with cutting down alcohol consumption in England?
Source: Drug Alcohol Depend. 2021 Oct 1;227:108938. doi: 10.1016/j.drugalcdep.2021.108938 (PMC8504198; doi:10.1016/j.drugalcdep.2021.108938)
Supplement: Supplementary file 1 [file mmc1.doc]

**Has the increased participation in Dry January been associated with cutting down on alcohol in England?**

Philippa Case ^1^

^1^ Department of Epidemiology & Public Health, University College London, WC1E 6BT, UK

Colin Angus ^2^, Frank De Vocht ^3,4^, John Holmes ^2^, Susan Michie ^5^, Jamie Brown ^6^,

^2^ Sheffield Alcohol Research Group, School of Health and Related Research, University of Sheffield, UK

^3^ Population Health Sciences, University of Bristol, UK

^4^ NIHR Applied Research Collaboration West (ARC West)

^5^ Department of Clinical, Educational and Health Psychology, University College London, UK

^6^ Department of Behavioural Science and Health, University College London, UK

To submit to: Drug and Alcohol Dependence

Key words: Dry January, temporary abstinence, alcohol, media campaigns, harm reduction

Correspondence to:

Philippa Case, Department of Epidemiology & Public Health, University College London, 1-19 Torrington Place, London WC1E 6BT

Email: philippa.case.17@ucl.ac.uk

Statement of competing interests:

The ATS receives funding from the NIHR School for Public Health Research (SPHR1 and 2). SPHR is a partnership between universities. The views expressed are those of the authors(s) and not necessarily those of the NHS, NIHR, or Department of Health. No funders had any involvement in the design of the study, the analysis or interpretation of the data, the writing of the report, or the decision to submit the paper for publication. JB's salary is funded by a programme grant from Cancer Research UK (CRUK; C1417/A22962). PC is funded by the Medical Research Council’s Doctoral Training Programme.

**Supplementary data**

Figure S1: Time series of the proportion of adults reporting drinking monthly or less for March 2015 to January 2015 and March 2017 to January 2018


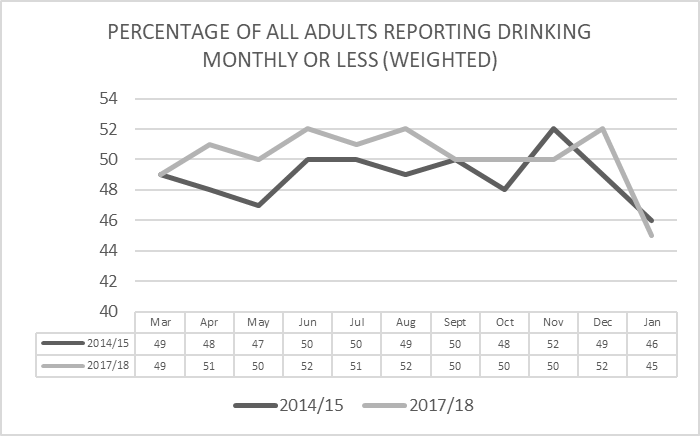


Figure S2: Time series mean weekly consumption in units for March 2015 to January 2015 and March 2017 to January 2018


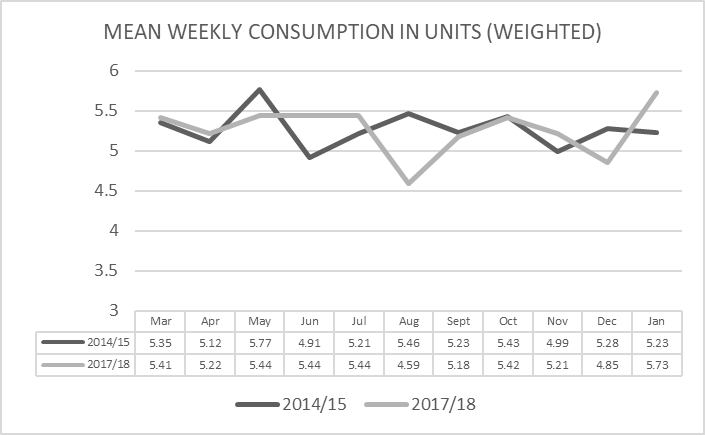


Planned Sensitivity Analyses

*2015/16 vs 2014/15*

*Primary outcomes:*

*i) Percentage of all adults reporting drinking* monthly or less frequently*.*

See Table S1. Percentage of adults drinking monthly or less frequently was lower in January than the preceding March-December in 2014/15 (46% vs 49%) but higher in January 2016 than the preceding March-December 2015 (50% vs 49%) as per Figure S3. There were no significant differences by month (January vs non-January) (OR 0.98, 95%CI 0.91-1.05) or by year (2014/15 vs 2017/18) (OR 1.00, 95%CI 0.96-1.04). The interaction between month and year was significant (OR 1.22, 95%CI 1.06-1.40) with the odds of reporting drinking monthly or less frequently being significantly lower in January vs non-January months in 2014/15 (OR 0.88, 95%CI 0.80-0.98) but with no significant difference between months in 2015/16 (OR 1.08, 95%CI 0.97-1.19).

*ii) Mean weekly alcohol consumption among adults who report drinking.*

See Table S2. Mean weekly alcohol consumption ranged from 4.8 units (SD 8.6) (January 2016) to 5.3 units (SD 9.8) (March-December 2014). Mean consumption did not differ significantly by month (β=-0.23, 95%CI=-0.58 to 0.11) or by year (β=-0.11, 95%CI=-0.30 to 0.09). The interaction between month and year was non-significant (β=-0.37, 95%CI=-1.05 to 0.32).

*Outcomes related to attempts to restrict alcohol consumption:*

*i) Percentage of at-risk drinkers reporting a current attempt to restrict alcohol consumption.*

See Table S3. Percentage of at-risk drinkers reporting an attempt to restrict consumption was higher in January than in other months in both 2014/15 (25% vs 20%) and 2015/16 (26% vs 20%) as per Figure S3. The odds of at-risk drinkers reporting a current attempt to restrict consumption was significantly higher in January vs non-January (OR 1.36, 95%CI 1.16-1.60) but there was no significant difference by year (2014/15 vs 2015/16) (OR 1.00, 95%CI 0.91-1.10). The interaction between month and year was non-significant (OR 1.06, 95%CI 0.77-1.46).

*ii) Percentage of at-risk drinkers citing Detox (e.g. Dry January) as a motive in their most recent attempt to restrict alcohol consumption.*

See Table S4. Percentage of at-risk drinkers citing *Detox (e.g. Dry January)* as a motive in their most recent attempt to restrict consumption was higher in January than in non-January months in both 2014/15 (13% vs 4%) and 2015/16 (9% vs 7%) as per Figure S5. The odds of at-risk drinkers citing *Detox (e.g. Dry January)* as a motive in the most recent attempt to restrict consumption were significantly higher in January vs non-January (OR 2.14, 95%CI 1.42-3.21) and significantly higher in 2015/16 vs 2014/15 (OR 1.55, 95%CI 1.11-2.15). The interaction between month and year was significant (OR 0.35, 95%CI 0.15-0.80), with the odds of citing *Detox (e.g. Dry January)* being significantly higher in January versus non-January in 2014/15 (OR 3.86, 95%CI 2.15-6.92), but with no significant difference between months in 2015/16 (OR 1.35, 95%CI 0.75-2.44).

*iii) Percentage of at-risk drinkers reporting use of a website or app to help restrict alcohol consumption in their most recent attempt.*

See Table S5. The percentage of at-risk drinkers reporting use of a website or app to help restrict alcohol consumption was the same in January vs other months in 2014/15 (2%) but was higher in January vs non-January months in 2015/16 (4% vs 3%). There were no significant differences by month (January vs non-January) (OR 1.40, 95%CI 0.68-2.87) or by year (2014/15 vs 2017/18) (OR 1.43, 95%CI 0.85-2.41). The interaction between month and year was non-significant (OR 1.94, 95%CI 0.40-9.50).

*2016/17 vs 2014/15*

*Primary outcomes:*

*i) Percentage of all adults reporting drinking monthly or less frequently.*

See Table S1. Percentage of adults drinking monthly or less frequently was lower in January than the preceding March-December in 2014/15 (46% vs 49%), but higher in January than the preceding March-December in 2016/17 (50% vs 49%) as per Figure S3. The odds of reporting drinking monthly or less frequently were significantly lower in January vs non-January (OR 0.87, 95%CI 0.81-0.94) and significantly higher in 2016/17 vs 2014/15 (OR 1.06, 95%CI 1.02-1.11). The interaction between month and year was non-significant (OR 0.97, 95%CI 0.84-1.12).

*iii) Mean weekly alcohol consumption among adults who report drinking.*

See Table S2. Mean weekly alcohol consumption ranged from 5.2 units (January 2015 (SD 8.8) and January 2017 (SD 9.1)) to 5.3 units (Mar-Dec 2014 (SD 9.8) and Mar-Dec 2016 (SD10.4)). Mean consumption did not differ significantly by month (β=-0.07, 95%CI=-0.43 to 0.28) or by year (β=0.05, 95%CI=-0.15 to 0.26). The interaction between month and year was non-significant (β=-0.05, 95%CI=-0.76 to 0.66).

*Outcomes related to attempts to restrict alcohol consumption:*

*i) Percentage of at-risk drinkers reporting a current attempt to restrict alcohol consumption.*

See Table S3. Percentage of adults reporting an attempt to restrict consumption was higher in January than in other months in both 2014/15 (26% vs 20%) and 2016/17 (22% vs 18%) as per Figure S4. The odds of at-risk drinkers reporting a current attempt to restrict alcohol consumption were significantly higher in January vs non-January (OR 1.30, 95%CI 1.11-1.53) and significantly lower in 2016/17 vs 2014/15 (OR 0.86, 95%CI 0.77-0.94). The interaction between month and year was non-significant (OR 0.97, 95%CI 0.70-1.34).

*ii) Percentage of at-risk drinkers citing Detox (e.g. Dry January) as a motive in their most recent attempt to restrict alcohol consumption.*

See Table S4. Percentage of at-risk drinkers citing *Detox (e.g. Dry January)* as a motive in their most recent attempt to restrict consumption was higher in January than in non-January months in both 2014/15 (13% vs 4%) and 2016/17 (21% vs 11%) as per Figure S5. The odds of at-risk drinkers citing *Detox (e.g. Dry January)* as a motive their most recent attempt to restrict alcohol consumptions was significantly higher in January vs non-January (OR 2.54, 95%CI 1.78-3.63) and significantly higher in 2016/17 vs 2014/15 (OR 2.55, 95%CI 1.86-3.48). The interaction between month and year was non-significant (OR 0.57, 95%CI 0.27-1.21).

*iii) Percentage of at-risk drinkers reporting use of a website or app to help restrict alcohol consumption in their most recent attempt.*

See Table S5. The percentage of at-risk drinkers reporting use of a website or app to help restrict alcohol consumption was the same in January vs other months in 2014/15 (2%) but was higher in January vs non-January months in 2016/17 (5% vs 3%). There was no significant difference in reported use of a website or app to help restrict alcohol consumption in January vs non-January months (OR 1.28, 95%CI 0.63-2.62), but the odds of reporting use of a website or app were significantly higher in 2016/17 vs 2014/15 (OR 1.83, 95%CI 1.11-3.04). The interaction between month and year was non-significant (OR 1.74, 95%CI 0.36-8.44).

Unplanned Sensitivity Analyses

1. *Percentage of adults reporting drinking never*

Due to the phrasing of the item *‘How often do you have a drink containing alcohol?*’ which asks participants about consumption during the past month, reporting drinking monthly or less frequently was selected as an outcome measure due to the possibility of some participants attempting to abstain in January still reporting consumption in December. To explore whether the results differed if participants reported drinking ‘never’ in the past month, the main analysis was re-run with ‘never’ as the outcome.

See Table S6. Percentage of adults drinking never was lower in January than the preceding March-December in both 2014/15 (28% vs 30%) and 2017/18 (28% vs 34%) as per Figure S6. The interaction between month and year was significant (OR 0.81, 95%CI 0.69-0.94), with the odds of reporting drinking never in the past month being significantly less in January versus non-January months in 2017/18 (OR 0.75, 95%CI 0.68-0.84) and no significant difference by month in 2014/15 (OR 0.94, 95%CI 0.84-1.05).

1. *Percentage of adults reporting drinking monthly or less frequently in February vs March-December*

To establish whether the unexpected result for percentage of people reporting drinking monthly or less frequently was a product of reporting error, due to participants being asked to report on drinking in the past month, the analysis for RQ1 was re-run using February data instead of January data.

See Table S7 for sample sizes. Percentage of adults drinking monthly or less frequently was the same in February as in the preceding March-December in both 2014/15 (49%) and 2017/18 (51%) as per Figure S7. The interaction between month and year was non-significant (OR 1.01, 95%CI 0.97-1.16).

**Tables and Figures: Sensitivity Analyses**

Planned Sensitivity Analyses

Table S1: Planned sensitivity analyses - Percentage of all adults reporting drinking monthly or less frequently in the last 6 months

|  | All drinkers | |
| --- | --- | --- |
|  | Drinking monthly or less frequently | Drinking more than monthly |
| **January 2015** |  |  |
| Unweighted N (%) | 804 (48) | 861 (52) |
| Weighted N (%) | 766 (46) | 901 (54) |
| **March-December 2014** |  |  |
| Unweighted N (%) | 8706 (52) | 8077 (48) |
| Weighted N (%) | 8227 (49) | 8562 (51) |
| **January 2016** |  |  |
| Unweighted N (%) | 901 (53) | 811 (47) |
| Weighted N (%) | 863 (50) | 849 (50) |
| **March-December 2015** |  |  |
| Unweighted N (%) | 8706 (52) | 7989 (48) |
| Weighted N (%) | 8114 (49) | 8586 (51) |
| **January 2017** |  |  |
| Unweighted N (%) | 979 (48) | 861 (52) |
| Weighted N (%) | 777 (47) | 881 (53) |
| **March-December 2016** |  |  |
| Unweighted N (%) | 8793 (52) | 8204 (48) |
| Weighted N (%) | 8609 (51) | 8393 (49) |
| **January 2018** |  |  |
| Unweighted N (%) | 739 (43) | 967 (57) |
| Weighted N (%) | 772 (45) | 967 (55) |
| **March-December 2017** |  |  |
| Unweighted N (%) | 8549 (50) | 8439 (50) |
| Weighted N (%) | 8599 (51) | 8391 (49) |

Figure S3: Planned sensitivity analyses – Percentage of all adults reporting drinking monthly or less frequently in past month (weighted)


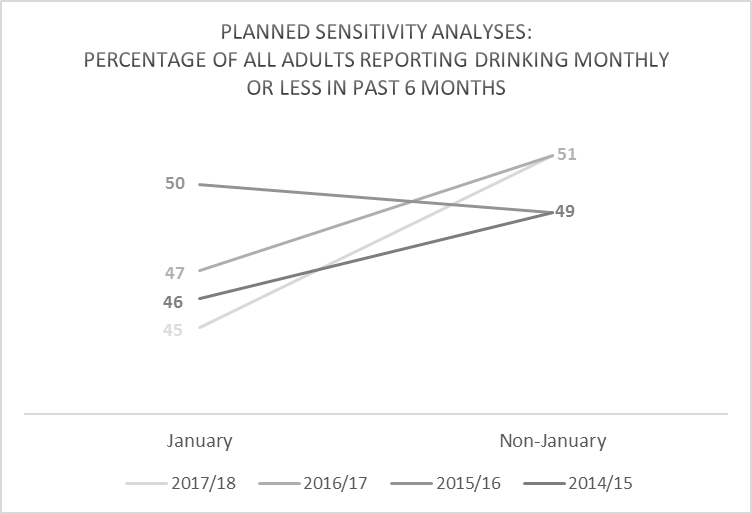


Table S2: Planned sensitivity analyses - Mean weekly alcohol consumption among adults who report drinking

|  | Adult drinkers | |
| --- | --- | --- |
|  | Mean consumption (units) | No. observations |
| **January 2015** |  |  |
| Unweighted (SD) | 5.3 (9.4) | 1661 |
| Weighted (SD) | 5.2 (8.8) | 1661 |
| **March-December 2014** |  |  |
| Unweighted (SD) | 5.1 (10.0) | 16,732 |
| Weighted (SD) | 5.3 (9.8) | 16,732 |
| **January 2016** |  |  |
| Unweighted (SD) | 4.7 (8.8) | 1707 |
| Weighted (SD) | 4.8 (8.6) | 1707 |
| **March-December 2015** |  |  |
| Unweighted (SD) | 5.0 (9.9) | 16,661 |
| Weighted (SD) | 5.2 (9.8) | 16,661 |
| **January 2017** |  |  |
| Unweighted (SD) | 5.3 (9.4) | 1655 |
| Weighted (SD) | 5.2 (9.1) | 1655 |
| **March-December 2016** |  |  |
| Unweighted (SD) | 5.3 (10.3) | 16,967 |
| Weighted (SD) | 5.3 (10.4) | 16,967 |
| **January 2018** |  |  |
| Unweighted | 6.2 (11.1) | 1706 |
| Weighted | 5.7 (10.3) | 1706 |
| **March-December 2017** |  |  |
| Unweighted | 5.3 (10.0) | 16,960 |
| Weighted | 5.2 (9.9) | 16,960 |

Table S3: Planned sensitivity analyses - Percentage of at-risk drinkers reporting a current attempt to restrict alcohol consumption

|  | At-risk drinkers attempting to restrict alcohol consumption | |
| --- | --- | --- |
|  | Yes | No |
| **January 2015** |  |  |
| Unweighted N (%) | 88 (20) | 342 (80) |
| Weighted N (%) | 112 (25) | 330 (75) |
| **March-December 2014** |  |  |
| Unweighted N (%) | 845 (20) | 3366 (80) |
| Weighted N (%) | 912 (20) | 3543 (80) |
| **January 2016** |  |  |
| Unweighted N (%) | 106 (26) | 300 (74) |
| Weighted N (%) | 115 (26) | 320 (74) |
| **March-December 2015** |  |  |
| Unweighted N (%) | 812 (20) | 3292 (80) |
| Weighted N (%) | 899 (20) | 3511 (80) |
| **January 2017** |  |  |
| Unweighted N (%) | 104 (23) | 346 (77) |
| Weighted N (%) | 103 (22) | 363 (78) |
| **March-December 2016** |  |  |
| Unweighted N (%) | 799 (18) | 3649 (82) |
| Weighted N (%) | 843 (18) | 3816 (82) |
| **January 2018** |  |  |
| Unweighted N (%) | 130 (26) | 369 (74) |
| Weighted N (%) | 135 (27) | 363 (73) |
| **March-December 2017** |  |  |
| Unweighted N (%) | 853 (19) | 3623 (81) |
| Weighted N (%) | 869 (19) | 3744 (81) |

Figure S4: Planned sensitivity analyses - Percentage of at-risk drinkers reporting a current attempt to restrict alcohol consumption (weighted)


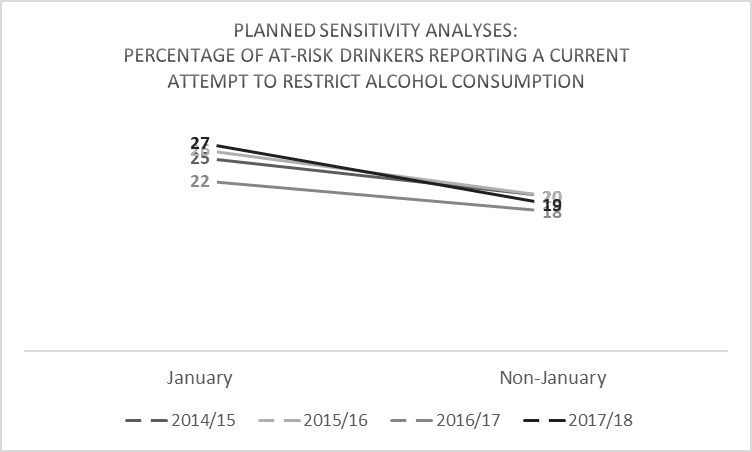


Table S4: Planned sensitivity analyses - Percentage of at-risk drinkers citing *Detox (e.g. Dry January)* as a motive in their most recent attempt to restrict alcohol consumption

|  | At-risk drinkers citing *Detox (e.g. Dry January)* | |
| --- | --- | --- |
|  | Yes | No |
| **January 2015** |  |  |
| Unweighted N (%) | 11 (9) | 106 (91) |
| Weighted N (%) | 18 (13) | 120 (87) |
| **March-December 2014** |  |  |
| Unweighted N (%) | 36 (4) | 952 (96) |
| Weighted N (%) | 40 (4) | 1024 (96) |
| **January 2016** |  |  |
| Unweighted N (%) | 12 (9) | 124 (91) |
| Weighted N (%) | 14 (9) | 136 (91) |
| **March-December 2015** |  |  |
| Unweighted N (%) | 76 (7) | 1048 (93) |
| Weighted N (%) | 88 (7) | 1150 (93) |
| **January 2017** |  |  |
| Unweighted N (%) | 25 (20) | 102 (80) |
| Weighted N (%) | 27 (21) | 102 (79) |
| **March-December 2016** |  |  |
| Unweighted N (%) | 116 (10) | 1023 (90) |
| Weighted N (%) | 126 (11) | 1066 (89) |
| **January 2018** |  |  |
| Unweighted N (%) | 28 (17) | 139 (83) |
| Weighted N (%) | 30 (18) | 137 (82) |
| **March-December 2017** |  |  |
| Unweighted N (%) | 128 (10) | 1112 (90) |
| Weighted N (%) | 137 (11) | 1126 (89) |

Figure S5: Planned sensitivity analyses - Percentage of at-risk drinkers citing *Detox (e.g. Dry January)* as a motive in their most recent attempt to restrict alcohol consumption (weighted)


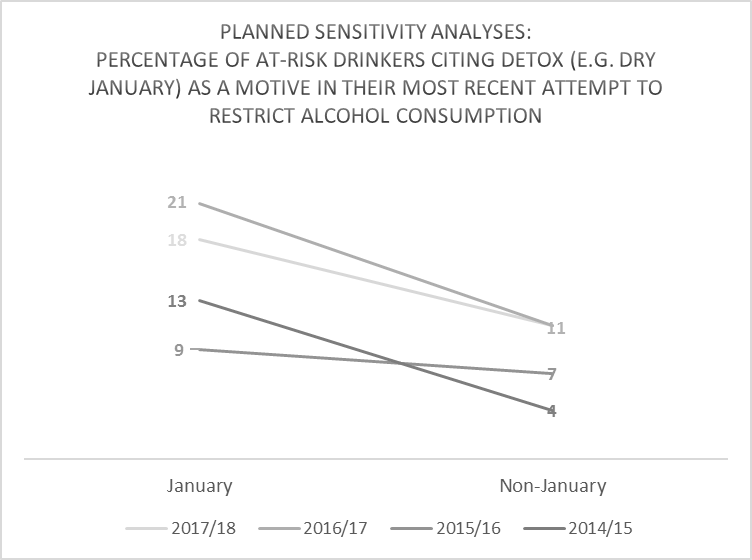


Table S5: Planned sensitivity analyses - Percentage of at-risk drinkers reporting use of a website or app to help restrict alcohol consumption in their most recent attempt

|  | At-risk drinkers reporting use of website or app | |
| --- | --- | --- |
|  | Yes | No |
| **January 2015** |  |  |
| Unweighted N (%) | 1 (1) | 116 (99) |
| Weighted N (%) | 2 (2) | 136 (98) |
| **March-December 2014** |  |  |
| Unweighted N (%) | 20 (2) | 969 (98) |
| Weighted N (%) | 21 (2) | 1045 (98) |
| **January 2016** |  |  |
| Unweighted N (%) | 5 (4) | 131 (96) |
| Weighted N (%) | 7 (4) | 143 (96) |
| **March-December 2015** |  |  |
| Unweighted N (%) | 27 (2) | 1097 (98) |
| Weighted N (%) | 31 (3) | 1206 (97) |
| **January 2017** |  |  |
| Unweighted N (%) | 7 (6) | 120 (94) |
| Weighted N (%) | 7 (5) | 122 (95) |
| **March-December 2016** |  |  |
| Unweighted N (%) | 34 (3) | 1105 (97) |
| Weighted N (%) | 39 (3) | 1153 (97) |
| **January 2018** |  |  |
| Unweighted N (%) | 4 (2) | 163 (98) |
| Weighted N (%) | 4 (2) | 164 (98) |
| **March-December 2017** |  |  |
| Unweighted N (%) | 37 (3) | 1203 (97) |
| Weighted N (%) | 39 (3) | 12325 (97) |

Unplanned Sensitivity Analyses

Table S6: Unplanned SA1: Percentage of all adults reporting drinking never in the past 6 months

|  | All drinkers | |
| --- | --- | --- |
|  | Drinking never | Drinking more than never |
| **January 2015** |  |  |
| Unweighted N (%) | 508 (31) | 1157 (69) |
| Weighted N (%) | 471 (28) | 1195 (72) |
| **March-December 2014** |  |  |
| Unweighted N (%) | 5447 (32) | 11,336 (68) |
| Weighted N (%) | 4976 (30) | 11,813 (70) |
| **January 2018** |  |  |
| Unweighted N (%) | 455 (27) | 1251 (73) |
| Weighted N (%) | 480 (28) | 1225 (72) |
| **March-December 2017** |  |  |
| Unweighted N (%) | 5790 (34) | 11,198 (66) |
| Weighted N (%) | 5812 (34) | 11,178 (66) |

Figure S6: Unplanned SA1: Percentage of all adults reporting drinking never in last 6 months

**
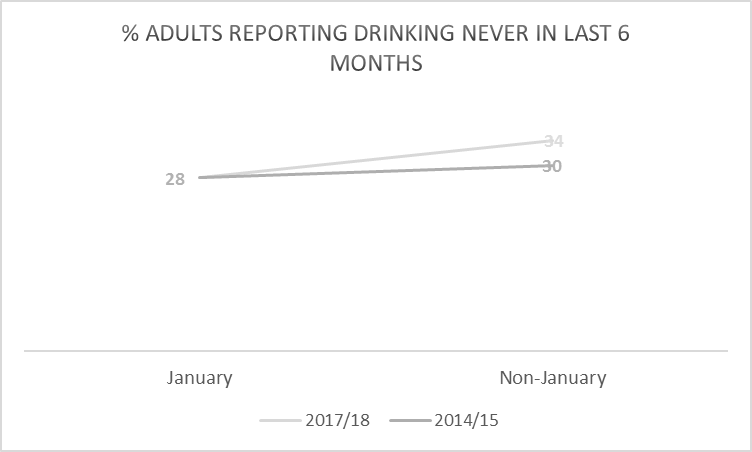
**

Table S7: Unplanned SA2: Percentage of all adults reporting drinking monthly or less frequently February vs March-December

|  | All drinkers | |
| --- | --- | --- |
|  | Drinking monthly or less frequently | Drinking more than monthly |
| **February 2015** |  |  |
| Unweighted N (%) | 856 (52) | 787 (48) |
| Weighted N (%) | 813 (49) | 830 (51) |
| **March-December 2014** |  |  |
| Unweighted N (%) | 8706 (52) | 8077 (48) |
| Weighted N (%) | 8227 (49) | 8562 (51) |
| **February 2018** |  |  |
| Unweighted N (%) | 836 (49) | 868 (51) |
| Weighted N (%) | 874 (51) | 831 (49) |
| **March-December 2017** |  |  |
| Unweighted N (%) | 8549 (50) | 8439 (50) |
| Weighted N (%) | 8599 (51) | 8391 (49) |

Figure S7: Unplanned SA2: Percentage of all adults reporting drinking monthly or less frequently February vs March-December

**
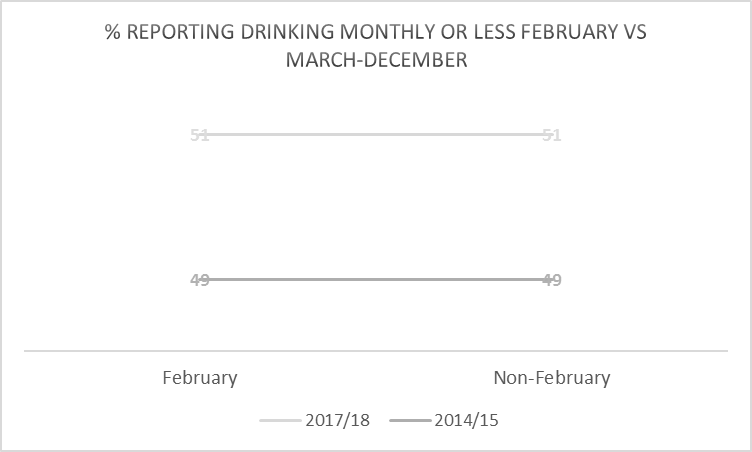
**
